# Genotoxicity and pharmacokinetic characterization of *Cereus jamacaru* ethanolic extract in rats

**DOI:** 10.1042/BSR20180672

**Published:** 2019-01-18

**Authors:** Iris Ucella de Medeiros, Rhoza Araújo de Medeiros, Raul Henandes Bortolin, Fernando Márlisson de Queiroz, Vivian Nogueira Silbiger, Stephan Pflugmacher, Aline Schwarz

**Affiliations:** 1 Post Graduation Program in Pharmaceutical Science, Federal University of Rio Grande do Norte, Natal, Brazil; 2 Pharmacy student, Federal University of Rio Grande do Norte, Natal, Brazil; 3 Department of Clinical and Toxicological Analysis, Federal University of Rio Grande do Norte, Natal, Brazil; 4 Department of Ecophysiology and Aquaculture, Leibniz-Institute of Freshwater Ecology and Inland Fisheries, Berlin, Germany

**Keywords:** folk medicine, Gene expression/regulation, Kidney/renal toxicology, Liver/hepatic toxicology, Pharmacokinetics

## Abstract

The effect of *Cereus jamacaru* ethanolic extract in rats was analyzed using genotoxicity assays and liver *ABCB1* and *CYP2D4* gene expression. The lyophilized extract of *C. jamacaru* cladodes was analyzed with LC–MS/MS. Male Wistar rats (*n*=36) were equally distributed into six groups that did (+) or did not (−) receive cyclophosphamide treatments: Control (−); Control (+); EXP 1 (−), and EXP 1 (+), both treated with 210 mg/kg of ethanolic extract; and EXP 2 (−) and EXP 2 (+), both treated with 420 mg/kg of ethanolic extract. After 30 d of treatment, body weight and food and water intake were monitored. Right femur of the rats and spinal canal fluid were harvested and used for genotoxicity assays, and the liver samples were used for gene expression studies. The phytochemical analysis identified novel compounds. Animals treated with *C. jamacaru* showed lower body weight and food ingestion compared to controls (*P*<0.05). The genotoxicity assay showed an absence of ethanolic extract cytotoxicity. *CYP2D4* expression was higher in EXP 2 groups compared with EXP 1 (−) group (*P*<0.05). *ABCB1A* expression was higher in negative groups compared with the positive groups. These results indicated a new phytochemical characterization of *C. jamacaru* and its effect on food ingestion and body weight gain. Moreover, the genotoxicity assay suggested that *C. jamacaru* ethanolic extract treatment presents significant intrinsic genotoxic potential and the enhanced expression of *ABCB1* and *CYP2D4* on *C. jamacaru* extract treatment suggests a role of the efflux transporter and microsomal enzyme, respectively, in *C. jamacaru* pharmacokinetics.

## Introduction

*Cereus jamacaru* DC, popularly known as ‘mandacaru’, is a representative species of the Cactaceae family and found in the Caatinga vegetation in Brazil [1]. *C. jamacaru* cladodes are used as diuretics and to lower the arterial blood pressure in traditional medicine. The syrup obtained from all parts of the plant is used to treat ulcers and respiratory tract affections, such as coughs and bronchitis, as well as to prevent scurvy [2–4]. However, although *C. jamacaru* is applied broadly in traditional medicine, there is limited information regarding its bioactive compounds.

Previous studies have shown that a wide range of compounds, including phenylethylamine alkaloids, tyramine, hordenine, mescaline, and lophophorine [5], as well as steroidal compounds, such as ß-sitosterol [6], are present in cacti. Brhun & Lindgren (1976) detected tyramine and 2-hydroxyphenylethylamine in *C. mandacaru* fresh cladodes [7]. A study of *C. jamacaru* seeds found that they contained a methionine-rich protein []. Additionally, phenylethylamine alkaloids, *N*-methyltyramine, tyramine, and hordenine were detected through liquid chromatography–UV (LC–UV) in crude ethanol extracts obtained from *C. jamacaru* cladodes [8].

However, to the best our knowledge, there is no evidence of the pharmacokinetic, pharmacodynamic, and toxicological properties of *C. jamacaru*.

The expression of individual CYPs is regulated by both endogenous and xenobiotic factors, including drugs and natural compounds. During drug development, in order to avoid undesirable drug–drug/herb interactions that may lead to changes in the rate of drug metabolism and potentially contribute to drug toxicity, it is helpful to acquire a preliminary understanding of the metabolism of a new chemical compound and its affinity for certain metabolic enzymes [9]. Moreover, transporter-mediated efflux of exogenous compounds and metabolites is an important cellular defense mechanism and plays a central role in the barrier and excretory functions in tissues, such as the intestinal mucosa, blood–brain and blood–testis barriers, renal proximal tubules, and liver. Several members of the ATP-binding cassette (ABC) transporter superfamily, including multidrug resistance protein 1 (MDR1/P-glycoprotein; ABCB1), breast cancer resistance protein (BCRP; ABCG2), and several members of the multidrug resistance-associated protein family (MRP; ABCC), are highly expressed in these barrier tissues, and their importance for drug efficacy is acknowledged [10,11].

Therefore, considering the lack of pharmacological and toxicological information about *C. jamacaru*, the present study aimed to investigate the effect of *C. jamacaru* ethanolic extracts in rats using genotoxicity assays and analyze the liver mRNA expression of *ABCB1* and *CYP2D4*.

## Materials and methods

### Plant and extract preparation

*C. jamacaru* cladodes used in the present study were collected in May and June 2010 in Natal (Rio Grande do Norte, Northeast Brazil). A representative specimen was deposited at the Herbarium of Universidade Federal do Rio Grande do Norte (registration number 4802). The ethanol extract obtainment was in accordance previous studies [12–15], with adaptations. Following thorn removal, fresh cladodes were sliced, dried at 40–50°C in a drying oven, and milled. A crude ethanol extract was obtained by macerating 1500 g dried milled cladodes in 5 L ethanol 96 GL for 7 d. After filtration, the solution obtained was concentrated under reduced pressure to produce the ethanol extract. The used milled cladodes (1500 g) yielded 26.2 g ethanol extract that was partially lyophilized (∼ 5 g).

### Extract characterization

Liquid chromatography tandem mass spectrometry (LC–MS/MS) for analysis of *C. jamacaru* lyophilized extract was performed according to Peuthert A. and Pflugmache S. (2010) [16]. The analysis was achieved using a quadrupole Applied Biosystems 3500 system (Applied Biosystems, Darmstadt, Germany) with the Agilent 1200 HPLC system consisting of a quaternary pump with a vacuum degasser, a temperature-controlled column compartment, an auto-sampler, and a photodiode detector (Agilent Technologies, Palo Alto, U.S.A.). An Agilent Eclipse Plus C18 (RP18) 4.6 × 50 mm 5-mm column run with a gradient of 15% of solution A (acetonitrile containing 0.1% (v/v) trifluoroacetic acid) and 85% of solution B (Milli-Q water containing 0.1% trifluoroacetic acid) over 20 min at a flow rate of 200 µl/min was used. The column temperature was set to 40°C and the injection volume was 10 ml. Electrospray ionization in multiple-reaction-monitoring (MRM) mode was performed in positive mode. In MRM mode, quadrupole 1 was fixed at a set parent ion, quadrupole 2 was used as a collision chamber to induce fragmentation, and quadrupole 3 was fixed at a set daughter ion. MRM was the preferred mode for quantification since it usually achieves the best possible specificity and signal-to-noise ratio. The desolvation gas (N2) was set to 85 psi and the collision gas (Ar) was tuned to a pressure of 20 psi. The source temperature was set to 600°C. For the electrospray source, the capillary voltage was set to 5.5 kV. Data were analyzed with the LightSight software for simultaneous metabolite identification (AB SCIEX, Toronto, Ontario and Foster City, California) [17–21].

### Animals

All animal experiments and protocols were approved by the Institutional Animal Research Ethics Committee (under Protocol No. 031/2012). All procedures were carried out in strict accordance with the recommendations from the Guide for the Care and Use of Laboratory Animals from the National Institutes of Health [22].

A total of 36 male Wistar rats (weight 200 g) were obtained from the animal care facility at the Federal University of Rio Grande do Norte, Brazil. The animals were housed under standard conditions (12-h light/dark cycle, a temperature of 22–24°C, and relative humidity of 50–60%) with food and water *ad libitum* during the entire experimental period. After 1 week of acclimatization before the experimental procedures, the animals were randomly assigned and equally distributed (*n*=6 each group) into the following groups, which did (+) or did not (−) receive cyclophosphamide treatments: Control (−); Control (+); EXP 1 (−), treated with 210 mg/kg of ethanolic extract; EXP 1 (+), treated with 210 mg/kg of ethanolic extract and cyclophosphamide; EXP 2 (−), treated with 420 mg/kg of ethanolic extract; and EXP 2 (+), treated with 420 mg/kg of ethanolic extract and cyclophosphamide.

The cyclophosphamide (Sigma Chemical Company Inc., St Louis, MO, U.S.A.) was administered in all animals from Control (+), EXP 1 (+), and EXP 2 (+) groups through a single intraperitoneal injection only at 29 d of experiment period. The cyclophosphamide solution was prepared using a freshly prepared in saline buffer vehicle at a dose of 50 mg/kg. Equal volumes of the vehicle were injected into the animals in the Control (−), EXP 1 (−), and EXP 2 (−) groups. The control groups received the vehicle used in *C. jamacaru* ethanolic extract, throughout the experimental period of 30 d. Body weight and food and water intake were monitored during all experimental period.

### Sample collection and modulating effects of *C. jamacaru* extract on the genotoxicity of cyclophosphamide

After 30 d of treatment, fasting animals were killed using a lethal dose of thiopental (100 mg/kg). Right femur bones were harvested, and the spinal canal was used for the assays. Briefly, the cells from spinal canal were obtained by washing with 1 ml of 0.9% saline buffer. After centrifugation procedures, the bone marrow cells were stained with May–Grunwald–Giemsa stain (Merck), modified by Rosenfeld for analyzing of polychromatic erythrocytes (PCE) and normochromic erythrocytes (NCE). Modulating effects of *C. jamacaru* extract on the genotoxicity of cyclophosphamide were evaluated using an optical microscope with a 100× magnification ocular lens. Results were expressed as the number of micronuclei PCE (MNPCE) in 1000 PCE. In addition, PCE ratio [PCE/(PCE + NCE)] was also calculated by counting 200 erythrocytes, for detecting the possibility of cytotoxicity. This *in vivo* Micronucleus Test (MN) in rat bone marrow is frequently used to detect clastogenic agents (which break chromosomes) and aneugenic agents (which induce aneuploidy or abnormal chromosome segregation due to mitotic spindle dysfunction) [23,24].

### RNA extraction and reverse transcriptase quantitative polymerase chain reaction (RT-qPCR)

The liver of each animal was previously stabilized by RNAlater®-ICE Frozen Tissue (Ambion, Austin, U.S.A.) and pulverized, and the total RNA was extracted using QIAamp® RNA Blood mini kit (Qiagen, Valencia, CA, U.S.A.). RNA integrity was assessed by electrophoresis in 1.0% agarose gels with MOPS [3-(*N*-morpholine)propanesulfonic acid] buffer, and RNA was quantitated using a NanoDrop ND-1000 spectrophotometer (Thermo Scientific, Wilmington, DE, U.S.A.) and stored at −80°C. cDNA was synthesized using 500 ng of total RNA and the High Capacity cDNA Reverse Transcription Kit (Applied Biosystems, Foster City, CA, U.S.A.), according to the manufacturer’s protocol, in a MyCycler thermal cycler (Bio-Rad, Philadelphia, PA, U.S.A.). cDNA obtained was stored at −20°C for later use in the RT-qPCR expression assays. RT-qPCR was performed using the TaqMan assay with the genes *ABCB1A* (Rn01639253_m1), *CYP2D4* (Rn01504629_m1), and glyceraldehyde-3-phosphate dehydrogenase (GAPDH, Rn01462661_g1) (Applied Biosystems). GAPDH was used as the reference gene. PCR assays were carried out in 96-well plates using a 7500 Fast Real-Time PCR System (Applied Biosystems). Relative expression was calculated using the 2^−ΔΔ*C*^_T_ method [25], and the results were presented as fold change compared with the mean values of the negative control group normalized to *GAPDH* expression, for which *C*_T_ did not show significant variations between the control and experimental groups.

### Statistical analysis

Statistical analyses were performed with Graph Pad PRISM software version 5.0 (GraphPad Software Inc., San Diego, CA, U.S.A.). For all mRNA relative expression data, we used the nonparametric Kruskal–Wallis and Mann–Whitney test for pairwise comparisons versus the control. The (anti)genotoxicity, cytotoxicity, body weight, and food and water intake data were analyzed by ANOVA and the post-hoc Holm-Sidak *t*-test for multiple comparisons; values *P*<0.05 were considered statistically significant.

## Results

### *C. jamacaru* extract characterization

**Figure 1:**
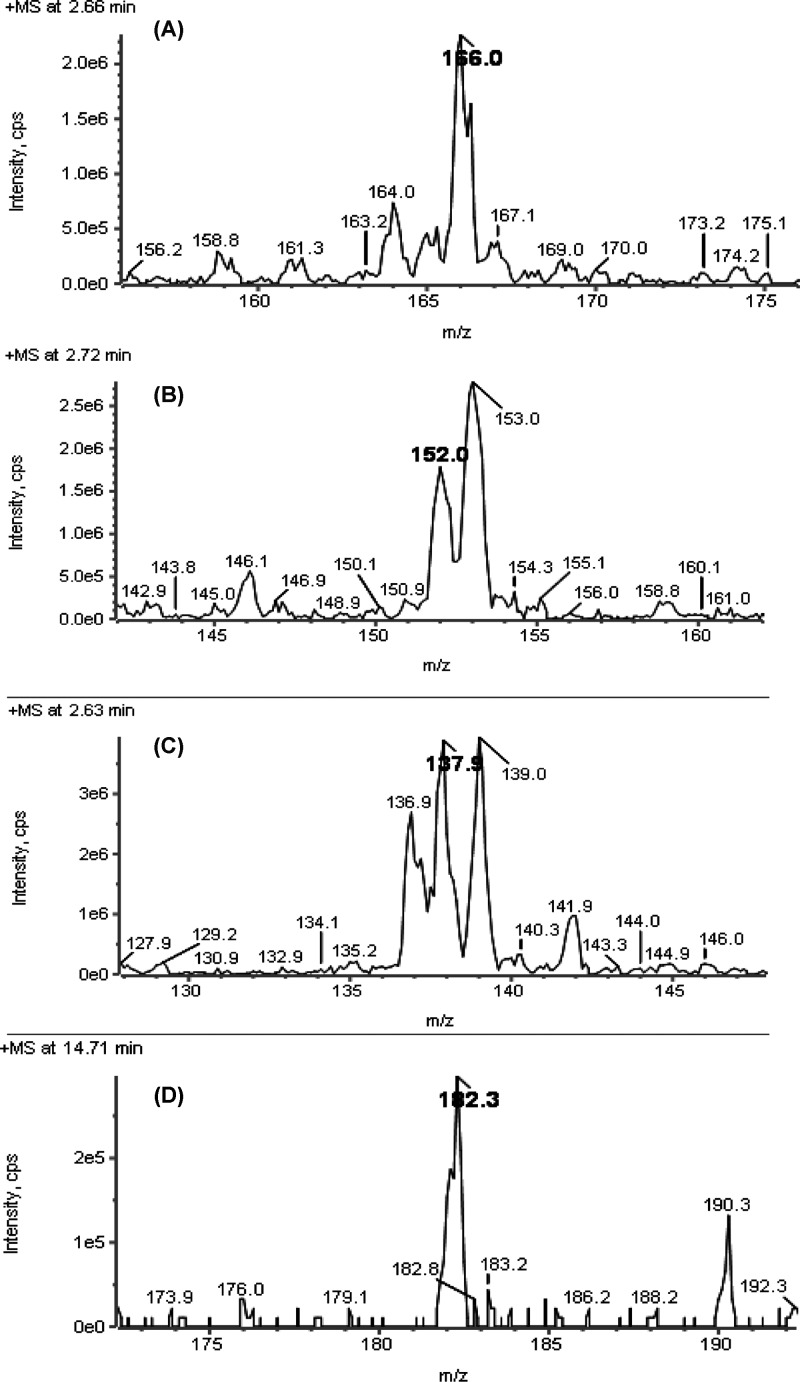
Mass spectrum analysis of *C. jamacaru* ethanolic extract (**A**) Hordenine; (**B**) N-methyltyramine; (**C**) Tyramine; (**D**) Tyrosine.

Phytochemical analysis performed by LC–MS/MS identified the phenethylamine alkaloids, hordenine, tyramine, *N*-methyltyramine, and tyrosine in the ethanolic extract (Figure 1). Other active compounds such as oleic acid, acetic acid, camphor, cysteine, geranyl acetone, corilagin, benzoic acid, cinnamic acid, 1,2-benzoquinone, anthraquinone, chloranil, hydroquinone, phenol, and β-sitosterol were also detected (Table 1). Our analysis detected several novel compounds in *C. jamacaru* cladodes, such as geranyl acetone, benzoquinone, anthraquinone, phenol, cinnamic acid, and valeric acid, indicating that cladodes produce several compounds that have potentially diverse metabolic, anti-inflammatory, calmative, anti-cancer, and chemotoxic properties.

**Table 1:** Phytochemical analysis of *C. jamacaru* ethanolic extract by LC–MS/MS

Compound - molecular structure/weight	Ethanol extract
Corilagin (gallotanine), C_27_ H_24_O_18_ /636.5	D
Benzoic acid, C_6_H_5_COOH/122.1	D
Arachidic acid, C_20_H_40_O_2_/312.5	D
Palmitic acid, C_16_H_32_O_2_/256	D
Oleic acid, C_18_H_34_O_2_/282	D
Cinnamic acid, C_9_H_8_O_2_/148.1	D
Stearic acid, C_18_H_36_O_2_/284.5	D
Caprylic acid, C_8_H_16_O_2_/144.2	D
Myristic acid, C_14_H_28_O_2_/228.4	D
Valeric acid, C_5_H_10_O_2_/102.1	D
Propionic acid, C_3_H_6_O_2_/74.1	D
Acetic acid, C_2_H_4_O_2_/60.1	D
1,2-Benzoquinone, C_6_H_4_O_2_/108.1	D
Anthraquinone, C_14_H_8_O_2_/208.2	D
Chloranil, C_6_Cl_4_O_2_/245.9	D
Hydroquinone, C_6_H_4_(OH)_2_/110.1	D
Phenol, C_6_H_6_O_1_/94.1	D
Camphor, C_10_H_16_O/152.2	D
Cysteine, C_3_H_7_NO_2_S/121.2	D
Geranylacetone, C_13_H_22_O/194	D
Hordenine, C_10_H_15_NO/165	D
Tyramine, C_8_H_11_NO/137	D
N-methyltyramine, C_9_H_14_NO/151	D
β-Sitosterol, C_29_H_50_O/414	D
Tyrosine, C_9_H_11_NO_3_/181.2	D
E-guggulsterone, C_21_H_28_O_2_/313.3	D

Lyophilized extract from *C. jamacaru* cladode was used for phytochemical characterization. Data were compared using the LightSight database program for metabolic identification. MRM mode: quadrupole 1 was fixed at a set parent ion; quadrupole 2 was used as a collision chamber to induce fragmentation; quadrupole 3 was fixed at a set daughter ion.

### Body weight and food and water intake

The animals treated with the *C. jamacaru* ethanolic extract showed lower body weight (EXP 1 (−) plus EXP 1 (+) and EXP 2 (−) plus EXP 2 (+)) compared to those in the control groups (control (−) plus control (+) *P*<0.05), indicating a lower body weight gain in animals treated with a high dose of the ethanolic extract (Table 2). Moreover, food intake was lower in animals treated with *C. jamacaru* ethanolic extracts when compared to that in the control animals (*P*<0.05). The water intake was not significantly different between the treatment groups.

**Table 2:** Body weight gain and food and water intake

	Control (−) plus control (+)	EXP1 (−) plus EXP1 (+)	EXP2 (−) plus EXP2 (+)	*P-value*
Body weight gain	42.83 ± 4.43	16.33 ± 7.32*	11.33 ± 4.15*	0.001
Food intake	38.25 ± 0.58	33.60 ± 0.79*	33.17 ± 0.53*	< 0.001
Water intake	65.00 ± 5.18	58.32 ± 1.78	65.00 ± 2.32	>0.05

Data are presented as means ± standard deviation of groups: Control (−), Control (+), healthy animals treated with cyclophosphamide, Exp 1 (−), healthy animals treated with 210 mg/kg of ethanolic extract, EXP 1 (+), healthy animals treated with 210 mg/kg of ethanolic extract plus cyclophosphamide, EXP 2 (−), healthy animals treated with 420 mg/kg of ethanolic extract, and EXP 2 (+), healthy animals treated with 420 mg/kg of ethanolic extract plus cyclophosphamide. ANOVA and the post-hoc Holm–Sidak *t*-test for multiple comparisons; *P*<0.05 was considered significant. *P<0,001 compared with control (−) plus control (+).

### Modulating effects of *C. jamacaru* extract on the genotoxicity of cyclophosphamide

The modulating genotoxicity assay showed, as expected, an increased number of micronucleated bone marrow cells in animals treated with cyclophosphamide: 1.64% in control (+) compared with 0.23% in control (−); 0.81% on EXP 1 (+) compared with 0.45% EXP 1 (−); and 1.74% on EXP 2 (+) compared with 1.28% on EXP 2 (−) (Table 3, *P*<0.05). However, comparing the negative groups, the incidence of micronucleated cells (MNPCE) increases with increasing dose of extract, with the difference attaining statistical significance at the high dose (EXP 2 (−)). The incidence of MNPCE in the control (−), EXP 1 (−), and EXP 2 (−) (i.e., control through high-dose groups) was 23.84, 45.17, and 128.81, respectively. These results indicate the genotoxic potential of the extract. Based on the PCE ratio parameter, the data obtained in the negative experimental groups when compared with the negative control group suggest an absence of cytotoxicity. In addition, the ethanolic extract was not able to neutralize clastogenicity induced by cyclophosphamide in treated rats but some attenuating effect on cyclophosphamide-induced genotoxicity in the low-dose group (e.g., EXP 1+ compared with control+) was observed.

**Table 3:** (Anti)Genotoxicity analysis of animals treated with *C. jamacaru* ethanolic extract and/or cyclophosphamide

	Control (+)	Control (−)	EXP1 (+)	EXP1 (−)	EXP2 (+)	EXP2 (−)	*F*
MNPCE	164.13 ± 16.81	23.84 ± 5.56 ^a/^***	81.73 ± 2.0^a/^**^, b/^***	45.17 ± 9.05 ^b/^***	173.88 ± 21.97^a/^***	128.81 ± 11.35^a/^***^, d/^*	23,057
PCE	199.17 ± 16.20	220.83 ± 16.75	246.67 ± 20.80	353.33 ± 28.13^a/^***^, b/^***^, c/^**	295.83 ± 22.30^a/^*^,b/^**	395.83 ± 25.18^a/^***^, b/^***^, d/^**	12,438
NCE	779.17 ± 16.75	800.83 ± 16.20	753.33 ± 20.80	646.67 ± 28.13^a/^***^, b/^***^, c/^**	704.17 ± 22.26 ^a/^**^,b/^*	604.17 ± 25.18^a/^***^, b/^***^, d/^**	12,438
PCE/NCE ratio	0.240 ± 0.02	0.277 ± 0.02	0.333 ± 0.4	0.562 ± 0.07 ^a/^***^, b/^***^, c/^**	0.427 ± 0.05^a/^*^,b/^*	0.671 ± 0.07 ^a/^***^, b/^***^, d/^**	10,891

NCE, normochromic erythrocytes; PCE, polychromatic erythrocytes; MNPCE, the number of micronuclei PCE. Data are presented as means ± standard deviation of groups: Control (−), Control (+), healthy animals treated with cyclophosphamide, Exp 1 (−), healthy animals treated with 210 mg/kg of ethanolic extract, EXP 1 (+), healthy animals treated with 210 mg/kg of ethanolic extract plus cyclophosphamide, EXP 2 (−), healthy animals treated with 420 mg/kg of ethanolic extract and EXP 2 (+), healthy animals treated with 420 mg/kg of ethanolic extract plus cyclophosphamide. ANOVA and the post-hoc Holm–Sidak *t*-test for multiple comparisons; *P*<0.05 was considered significant. a/*** *P*<0.001 compared with control (−) group; a/**P<0.01 compared with control (−) group; a/* *P*<0.05 compared with control (−) group; b/*** *P*<0.001 compared with control (+) group; b/** *P*<0.01 compared with control (+) group; b/* *P*<0.05 compared with control (+) group; c/** *P*<0.01 compared with EXP1 (+) group; d/** *P*<0.01 compared with EXP2 (+) group; d/* *P*<0.05 compared with EXP2 (+) group.

In addition, there were alterations observed in the liver, kidney, heart, pancreas, and spleen organ weight/body weight ratios between the control and treatment groups (Table 4). A histopathological study also indicated an absence of cellular morphological changes in animals treated with the extract or cyclophosphamide (data not shown).

**Table 4:** Organs/body weight ratios of animals treated with *C. jamacaru* ethanolic extracts and/or cyclophosphamide

	Control (−) plus control (+)	EXP1 (−) plus EXP1 (+)	EXP2 (−) plus EXP2 (+)
Liver	29.1 ± 0.463	29.8 ± 0.679	30.4 ± 1.020
Kidney	3.21 ± 0.0773	3.23 ± 0.199	3.46 ± 0.0883
Heart	3.21 ± 0.0949	3.28 ± 0.0944	3.43 ± 0.0494
Splenn	2.5 ± 0.218	2.22 ± 0.185	2.37 ± 0.204
Pancreas	1.4 ± 0.121	1.62 ± 0.206	1.61 ± 0.142

Data are presented as means ± standard deviation of groups: Control (−), Control (+), healthy animals treated with cyclophosphamide, Exp 1 (−), healthy animals treated with 210 mg/kg of ethanolic extract, EXP 1 (+), healthy animals treated with 210 mg/kg of ethanolic extract plus cyclophosphamide, EXP 2 (−), healthy animals treated with 420 mg/kg of ethanolic extract and EXP 2 (+), healthy animals treated with 420 mg/kg of ethanolic extract plus cyclophosphamide. ANOVA and the post-hoc Holm–Sidak *t*-test for multiple comparisons; *P*<0.05 was considered significant.

### *ABCB1A* and *CYP2D4* expression

**Figure 2:**
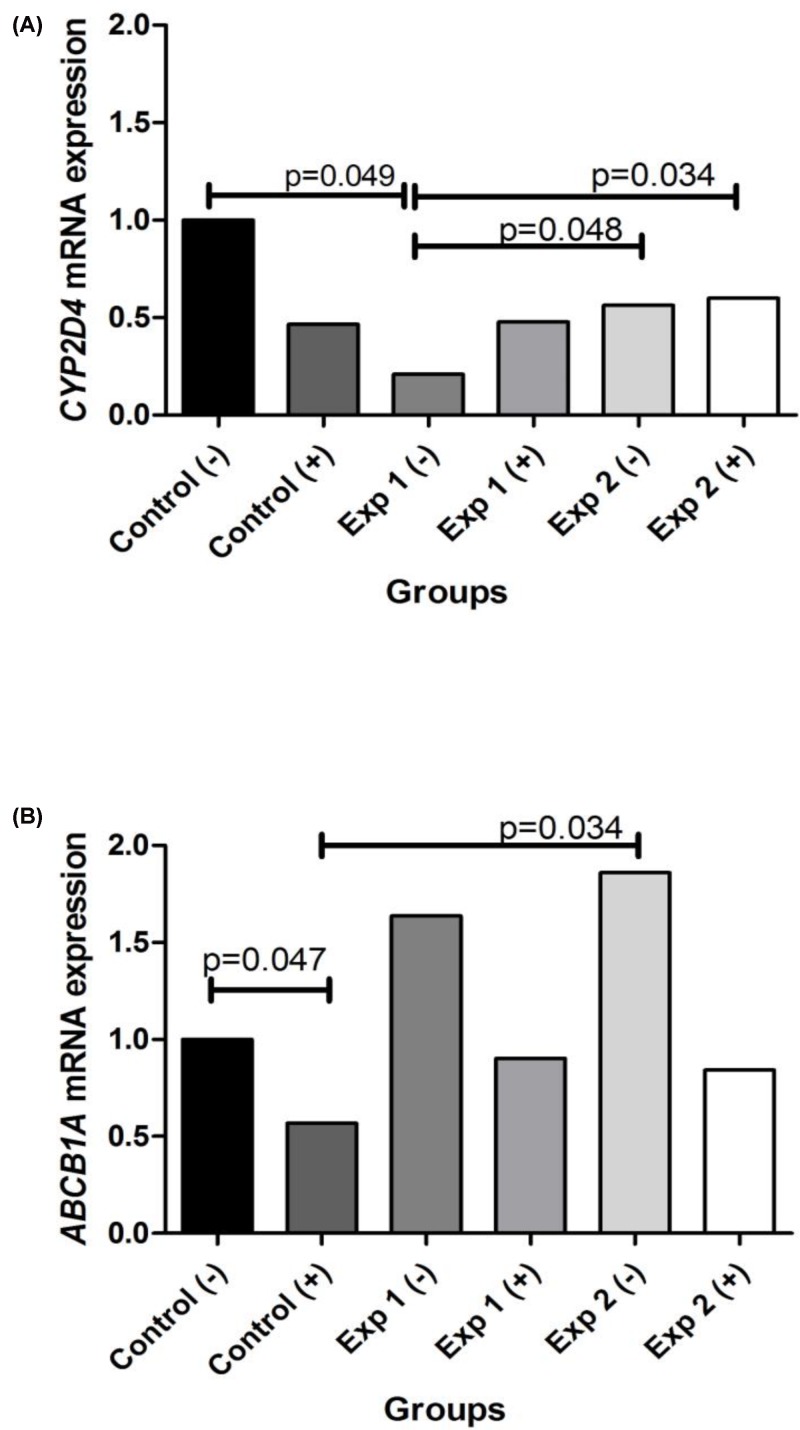
Relative mRNA expression quantification of ABCB1A and CYP2D4 in the liver CYP2D4 (**A**) and ABCB1A (**B**) mRNA expression in liver tissue of Control (−); Control (+), healthy animals treated with cyclophosphamide; EXP 1 (−), healthy animals treated with 210 mg/kg of ethanolic extract; EXP 1 (+), healthy animals treated with 210 mg/kg of ethanolic extract plus cyclophosphamide; EXP 2 (−), healthy animals treated with 420 mg/kg of ethanolic extract; and EXP 2 (+), healthy animals treated with 420 mg/kg of ethanolic extract plus cyclophosphamide. All data are expressed as fold-change compared with control (−) group values, normalized to GAPDH. Comparisons between groups were analyzed with Kruskal–Wallis ANOVA on Ranks and Dunn’s post-hoc. *P*<0.05 was considered significant.

The *CYP2D4* mRNA expression (Figure 2A) was reduced in the EXP 1 (−) group in comparison with that in the control (−) group (*P*=0.049). However, *CYP2D4* gene expression was higher in EXP 2 (−) and EXP 2 (+) groups compared with that in EXP 1 (−) (*P*=0.048 and 0.034, respectively). The expression of *ABCB1A* (Figure 2B) was reduced in the control (+) compared with that in the control (−) groups, while the expression was higher in the EXP 2 (−) group compared with that in control (+). Moreover, we observed a reduction in *ABCB1A* expression in all groups of animals that received a single dose of cyclophosphamide, despite the reduction has been not significant when all conditions were compared.

## Discussion

The phytochemical screen obtained by LC–MS/MS showed the presence of biologically active compounds including the phenylethylamine alkaloids hordenine, tyramine, *N*-methyltyramine, and tyrosine in the *C. jamacaru* extract. Although a few of these phenylethylamine compounds were previously detected by HPLC–UV [8], the present study detected several other compounds, which have been never before identified in *C. jamacaru*, such as geranyl acetone, benzoquinone, anthraquinone, phenol, cinnamic acid, and valeric acid. These new phytochemical compounds are important because there are limited data about *C. jamacaru* composition [7,8,26–28], and particularly because *C. jamacaru* is used as food for livestock during droughts and in the treatment of some diseases in traditional medicine [2–4,29]; these phytochemicals could provide information to better understand the mechanism of action and toxicity of *C. jamacaru*.

Phenylethylamines and 3,4-methylenedioxy-*N*-methylamphetamine (MDMA, ‘ecstasy’) are metabolized by the metabolic enzyme, CYP2D6 [30,31]. There are over 30 derivatives of phenylethylamine and tyramine in FDA-approved drugs, which are used in the treatment of anaphylaxis (epinephrine), asthma (formoterol), and depression (phenelzine) [32]. Hordenine stimulates norepinephrine, a hormone that mediates lipid release and can lead to body weight loss and inhibited gut movements, which contributes to decreased weight gain [33,34]. *N*-methyltyramine is a protoalkaloid isolated from various plant species, and it is assumed to be an adrenergic agonist with pharmacological properties similar to other structurally related biogenic amines, such as *p*-synephrine, *p*-octopamine, epinephrine, and norepinephrine, as well as ephedrine and amphetamine, which are phenylpropylamine derivatives [35]. Moreover, *p*-synephrine has been shown to reduce body weight in pre-clinical and clinical studies [33,36]. These data help us better understand phytochemical action and are consistent with our observations of reduced body weight gain in animals traded with *C. jamacaru* ethanolic extracts. Moreover, to the best of our knowledge, this is the first study to show a potential beneficial effect of *C. jamacaru* against obesity. Additional studies will be needed to confirm our results and the further use of *C. jamacaru* as a therapeutic drug treatment.

Information on genotoxicity is required irrespective of the extent of migration and resulting human exposure, in view of the theoretical lack of threshold for genotoxic events [37]. Among the genotoxicity tests recommended by international regulatory agencies and government institutions, the *in vivo* micronucleus test (MN) using rat bone marrow is widely assessed as one of the few tests required for the assessment and recording of new chemical and pharmaceutical products that are introduced into the world market [38,39]. This test is frequently used to detect clastogenic agents that break chromosomes and aneugenic agents that induce aneuploidy or abnormal chromosome segregation due to mitotic spindle dysfunction [40,41]. This method was initially developed in mouse bone marrow erythrocytes [42], but can also be performed in rats [43].

The cyclophosphamide used as positive control belongs to class of oxazaphosphorines and it is an alkylating agent extensively used as an anti-cancer chemotherapeutic agent. Cyclophosphamide has been extensively tested to induce dominant lethal mutation, DNA damage, and generation of free radicals or Reactive Oxygen Species *in vivo* as well. Free radicals due to their high chemical reactivity induce cellular damage in several ways. The most deleterious effects of cyclophosphamide free radicals *in vivo* were genotoxic activities including DNA damages, chromosome aberrations, sister chromatid exchanges, and gene mutations, which can lead to many pathological conditions including cancer [44–49].

In the present study, we did not observe a reduction in PCE ratio values for groups treated only with *C. jamacaru* ethanolic extracts when compared with the negative control, indicating an absence of cytotoxicity in the ethanolic extract treatments. However, the incidence of micronucleated cells (MNPCE) in rats not treated with cyclophosphamide increases with increasing dose of extract, with the difference attaining statistical significance at the high dose (EXP 2 (−)). The incidence of MNPCE in the control (−), EXP 1 (−), and EXP 2 (−) (i.e., control through high-dose groups) was 23.84, 45.17, and 128.81, respectively. The incidence (128.81) in EXP 2 (−) is more than 5-fold that in the respective control (−) group and in fact approaches that of the cyclophosphamide positive control (+) (164.13), suggesting significant intrinsic genotoxic potential. The ethanolic extract was not able to neutralize clastogenicity induced by cyclophosphamide.

The molecular analysis indicated a reduced expression of *CYP2D4* in animals treated with low doses of *C. jamacaru* ethanolic extract, while high doses of *C. jamacaru* ethanolic extract with or without cyclophosphamide treatment led to an increased expression of *CYP2D4* compared with low doses, suggesting a role of this CYP isoform in *C. jamacaru* ethanolic extract pharmacokinetics in male rats. In humans, the CYP2D enzymes are particularly important in drug metabolism, constituting 2–4% of the total hepatic CYPs and the CYP2D isoform is responsible for the metabolism of 20–25% of commonly prescribed drugs [50]. Particularly, for CYP2D4, previous studies that aimed to determine the effects of traumatic brain injury alone and with erythropoietin or anakinra treatment by analyzing gene expression of hepatic inflammatory proteins, drug-metabolizing enzymes, and transporters in a cortical contusion impact injury model showed that traumatic brain injury and erythropoietin treatment decreased the expression of *CYP2D4* mRNA, as well as the CYP2D4 protein [50].

*ABCB1A* expression increased in animals treated with *C. jamacaru* ethanolic extract, while in the presence of cyclophosphamide there was a reduction in *ABCB1A* expression, suggesting that transporter-mediated efflux of exogenous compounds and metabolites could be a component of *C. jamacaru* pharmacokinetics. Moreover, *ABCB1A* was down-regulated in cyclophosphamide-treated animals, which suggests a potential drug-phytochemical interaction between *C. jamacaru* extracts and cyclophosphamide and supports our genotoxicity results.

Transporters play a vital role in protecting cells from xenobiotics and altering the function of these transporters may lead to a severe physiological imbalance that results in a high level of toxicity. *P*-glycoprotein (P-gp; MDR1, ABCB1) is highly expressed in leukaemia, breast, ovarian, colon, kidney, adrenocortical, and hepatocellular cancers, and its overexpression is inversely correlated with poor clinical prognosis [51–53]. It is localized at the apical surface of cells and is highly expressed in the capillary endothelial cells that act as barriers at the blood–brain barrier, placental trophoblasts, the testes, intestines, the liver, kidneys, and the adrenal glands, suggesting that the physiological role of *P*-gp is to protect the body against xenobiotics and toxins [54,55].

In summary, we present a new phytochemical characterization of *C. jamacaru* that may be useful in describing its use as a herbal drug, particularly in the treatment of obesity, as indicated by our observations that treatment with *C. jamacaru* extracts reduced food ingestion and body weight gain in rats. However, the modulating genotoxicity assay suggests that *C. jamacaru* extract presents significant intrinsic genotoxic potential. This treatment also altered the expression of *ABCB1* and *CYP2D4*, suggesting that metabolic enzymes and transporters may contribute to the pharmacokinetic effects of *C. jamacaru*.

## Abbreviations

ABC: ATP-binding cassette
LC: liquid chromatography
MNPCE: micronuclei PCE
MRM: multiple-reaction-monitoring
MS: mass spectrometry
NCE: normochromic erythrocytes
PCE: polychromatic erythrocytes
P-gp: *P*-glycoprotein
